# DNA-binding transcription factor NF-1A negatively regulates JC virus multiplication

**DOI:** 10.1099/vir.0.2008/000059-0

**Published:** 2008-06

**Authors:** Veerasamy Ravichandran, Eugene O. Major

**Affiliations:** Laboratory of Molecular Medicine and Neuroscience, National Institute of Neurological Disorders and Stroke, National Institutes of Health, Bethesda, MD 20892, USA

## Abstract

JC virus (JCV) DNA replication occurs in the nuclei of infected cells. The level of JCV genome expression depends on nucleotide sequences in the viral regulatory region and their interaction with host-cell nuclear transcription factors. Our previous studies showed a higher level of NF-1X in JCV-permissive cells compared with the other members of the NF-1 family, NF-1A, B and C, which suggests that NF-1X plays a positive role in JCV multiplication. It remained unclear whether a reduction in the level of NF-1A, which is expressed abundantly in JCV-non-permissive cell types, leads to an increase in JCV multiplication. In this study, we show that downregulation of NF-1A expression in JCV-non-susceptible progenitor and HeLa cells results in a reversion to susceptibility for JCV multiplication. These data demonstrate that a higher level of NF-1A protein in JCV-non-permissive cell types, compared with the level of NF-1X, may be acting as a negative regulator at the JCV promoter to control JCV multiplication.

The human polyomavirus JC virus (JCV) is responsible for central nervous system (CNS) demyelination leading to progressive multifocal leukoencephalopathy (PML) (Astrom *et al.*, 1958). Serological studies suggest that approximately 70 % of the adult population worldwide have experienced primary JCV infection (Walker & Padgett, 1983; Hamilton *et al.*, 2000). PML almost invariably follows from reactivation of latent JCV in patients with compromised cellular immunity (Berger & Major, 1999), particularly in patients with HIV/AIDS, but also occurs in those with leukaemia, lymphoma and connective tissue diseases. Individuals that receive immunomodulatory therapy for autoimmune disorders may also develop PML (Yousry *et al.*, 2006). The molecular regulation of JCV expression limits the range of cell types that can serve as sites of JCV latency and reactivation (Imperiale & Major, 2007). The cellular host range of JCV depends upon a number of factors: attachment of the virus on a host cell, virus internalization and transport, uncoating and delivery of the viral genome, viral DNA transcription, viral protein propagation, virus assembly and virus release. Nuclear transcription factors, which play fundamental roles in regulating JCV multiplication, exist at the centre of these molecular pathways.

Host-cell nuclear transcription factors are required for the activation of the JCV promoter/enhancer (Nathanson *et al.*, 1997; Raj & Khalili, 1995). Nucleotide sequences that act as transcriptional promoters are located immediately upstream of the origin of DNA replication (Delbue *et al.*, 2005; Nathanson *et al.*, 1997). These sequences include the TATA box and binding sites for Sp1, YB1, sup2, Pur-*α* and NF-1. The NF-1 site is directly adjacent to an AP-1 or c-jun/c-fos site (Ciappi *et al.*, 1999; Ravichandran *et al.*, 2006). The nuclear transcription factor recognition sites are duplicated in the tandem repeat arrangement found in JCV isolates that support viral expression in permissible cells (Delbue *et al.*, 2005). These transcription factors are required for activation of the JCV promoter/enhancer. Cell-specific expression of JCV has been associated with several transcription factors, but the mechanisms through which the JCV promoter is controlled by these transcription factors are poorly understood.

The nuclear transcription factor NF-1 is an example of a cell-specific regulator of JCV promoter/enhancer activity (Chen & Khalili, 1995). The NF-1 transcription family of DNA-binding proteins (NF-1A, NF-1B, NF-1C and NF-1X) is encoded by four genes (Gronostajski, 2000; Kruse *et al.*, 1991; Kruse & Sippel, 1994; Sumner *et al.*, 1996). Many NF-1-binding sites are found in the JCV promoter/enhancer region (Amemiya *et al.*, 1992; Ciappi *et al.*, 1999; Jeang *et al.*, 1987; Tamura *et al.*, 1988). Products of the four NF-1 genes form homo- and heterodimers that bind to the canonical NF-1-binding site with apparently identical affinities (Gronostajski, 2000). Mutations in the NF-1-binding sites reduce JCV expression (Amemiya *et al.*, 1989). We have shown previously that the level of expression of NF-1 proteins differs in JCV-permissive and -non-permissive cells (Messam *et al.*, 2003). Human progenitor-derived astrocyte cells that support JCV infection expressed high levels of NF-1X when compared with the non-permissive cell line HeLa. JCV-susceptible cells showed higher levels of NF-1X and lower levels of NF-1A compared with JCV-non-susceptible cells.

While all NF-1 proteins appear to bind to the same DNA sequence, differences in function among NF-1 gene products have been observed in a number of systems. The promoters of several genes were shown to be activated by NF-1 proteins (Bisgrove *et al.*, 2000; Jones *et al.*, 1987; Shaul *et al.*, 1986). Genes that are negatively regulated by NF-1 also exist (Fazi *et al.*, 2005; Kleene *et al.*, 2001; Wong *et al.*, 2007). Since NF-1A is shown to function as a negative regulator of many genes, the current study aims to determine whether higher levels of NF-1A expressed in JCV-non-susceptible cells act as a negative regulator for JCV multiplication. We used different techniques to decrease the level of NF-1A in JCV-non-susceptible cell types and observed an increase in JCV multiplication. This indicates an essential negative regulatory role of NF-1A in JCV tropism.

Human brain-derived progenitor cells (referred to here as progenitors) were obtained from the telencephalon of an 8-week gestational fetal brain, in accordance with NIH guidelines as described previously (Messam *et al.*, 2003). Progenitor cultures grown at 10–40 % confluence were at least 98 % positive for nestin staining and did not express glial fibrillary acidic protein (GFAP). Differentiation of progenitors into an astrocytic lineage (referred to as progenitor-derived astrocytes; PDA) was initiated by culture medium substitution as described before (Messam & Major, 2000). HeLa cells were grown with MEM supplemented with 10 % fetal bovine serum. For induction of TGF-*β*1 signalling, cultures were treated with 5 ng recombinant TGF-*β*1 ml^−1^ (R&D Systems). Progenitor, PDA or HeLa cultures were exposed to JCV (Mad-4 variant) at 100 haemagglutination units (HAU) per 5×10^5^ cells in a minimal covering of appropriate serum-free medium. After overnight JCV exposure, cultures were washed and replenished with appropriate fresh media. All JCV-exposed cultures were processed 4 days after JCV exposure.

siRNA against NF-1A or NF-1B (Dharmacon) or control siRNA (SantaCruz) (final concentration of 10 nM) was used to transfect cells using appropriate nucleofector reagents (Amaxa, Inc.). Human miR-223 (hsa-miR-223; Dharmacon) was transfected into progenitor cells (final concentration 10 nM) using nucleofector reagent (Amaxa, Inc.). At 16 h post-transfection, the culture medium was replaced with fresh medium containing JCV at 100 HAU per 5×10^5^ cells. After 8 h of JCV exposure, viral medium was removed by aspiration, the cells were washed once and fresh medium was added.

Four days after the transfection (3 days after JCV exposure), nuclear fractions or whole-cell lysates were prepared for use in Western blot experiments with appropriate antibodies as described before (Ravichandran *et al.*, 2007). The antibodies used were: anti-JCV-VP-1 (rabbit polyclonal antibody developed in our laboratory), anti-JVC-VP-1 (monoclonal; Novocastra), anti-SV40 T-antigen (Oncogene), anti-*β*-actin (Sigma), anti-NF-1A (Geneka/Active Motif), anti-NF-1B (Geneka/Active Motif) and anti-NF-1X (Geneka/Active Motif). Bound primary antibodies were detected using either an anti-rabbit or anti-mouse horseradish peroxidase-conjugated secondary antibody combined with the SuperSignal West Pico Chemiluminescent substrate kit (Pierce), according to the manufacturer's protocol.

RT-PCR was performed as follows. Four days after the transfection of miR-223 into progenitor cells, total RNA was isolated using the RNeasy total RNA isolation system (Qiagen) followed by treatment for 60 min with DNase I [10 U (μg RNA)^−1^; Boehringer Mannheim]. First-strand cDNA synthesis was performed on 1 μg total RNA using oligo dT primers and Superscript II reverse transcriptase (Invitrogen). cDNA product (2 μl) served as the template for specific PCR amplification. The PCR primers for NFI-A were 5′-GTCAGCTTCACTTGGCTGGC (5′ primer) and 5′-AGCTTTATCTTTCCGTAACTTGGCCCGGATATCAAGGCCAAGTTACGGAAAGATATCG (3′ primer). PCR amplification consisted of 30 cycles of 30 s at 95 °C, 30 s at 55 °C and 1 min at 72 °C using a Perkin-Elmer 2400 thermal cycler. PCR products (20 μl) were subjected to 1.5 % agarose gel electrophoresis, stained with ethidium bromide and photographed.

Four days after the transfection of miR-223 into progenitor cells (3 days after JCV exposure) or 4 days after treatments with or without TGF-*β*1, HeLa cells grown with JCV on chamber slides were fixed with 4 % paraformaldehyde in PBS for 20 min at room temperature and permeabilized with 0.2 % Triton in PBS for 10 min at room temperature. Blocking, probing and visualization were done as described previously (Ravichandran *et al.*, 2007).

Because of the differentiation of JCV-non-susceptible progenitors into JCV-susceptible PDAs, we examined the role of NF-1 protein levels in these cell types. Differentiation of progenitor cells into PDA was accompanied by a gradual increase in the level of NF-1X. Notably, levels of NF-1A and NF-1B decreased (Fig. 1a[Fig f1]). Our observation that progenitor cells express very low levels of NF-1X protein supports our previous study, which showed an essential role of NF-1X protein in JCV multiplication.

The abundance of NF-1A and NF-1B in progenitor cells as well as other JCV-non-susceptible cells (i.e. HeLa cells, progenitor-derived neurons) raised the possibility of a negative regulatory role of NF-1A and NF-1B in these cell types. An earlier study showed that, following the addition of TGF-*β*1, JCV multiplication increased (Ravichandran *et al.*, 2007). As an extension of this observation, we also observed a decrease in NF-1A and NF-1B levels under these conditions in progenitor cells (Fig. 1b[Fig f1]). No increase in the level of NF-1X was observed, however (not shown). These results raised the possibility that NF-1A and/or NF-1B may be acting as negative regulators for JCV multiplication. To address the mechanism through which NF-1A and/or NF-1B suppresses the multiplication of JCV in non-susceptible cells, progenitors were transfected with siRNA corresponding to NF-1A or NF-1B. Interestingly, the NF-1A siRNA reduced the level of NF-1A in progenitor cells and increased JCV multiplication, as measured by the level of VP-1 protein. However, the reduction in NF-1B by NF-1B siRNA did not lead to an increase in JCV multiplication (Fig. 1c[Fig f1]). In addition, the reduction in the level of NF-1A led to an increase in NF-1B. Conversely, an increase in NF-1A led to a decrease in NF-1B.

To examine the negative role of NF-1A in JCV multiplication, another JCV-non-susceptible cell type, HeLa, was studied. Addition of TGF-*β*1 to HeLa in the presence of JCV showed increased JCV multiplication, as observed by increased VP-1 protein in immunofluorescence experiments (Fig. 2a[Fig f2]) as well as in a Western blot (Fig. 2b[Fig f2]). Further, the reduction of the level of NF-1A protein in HeLa cells by siRNA resulted in JCV multiplication (Fig. 2c[Fig f2]). Based on the observation by Fazi *et al.* (2005) that miR-223 regulates the NF-1A protein level, we transfected hsa-miR-223 into progenitor cells and found a decrease in the level of NF-1A protein without affecting the mRNA level. This reduction in NF-1A protein was accompanied by an increase in JCV multiplication, as measured by Western blot (Fig. 3a[Fig f3]) as well as immunofluorescence experiments (Fig. 3b[Fig f3]). Taken together, our data demonstrate that any means of reducing the abundance of NF-1A protein in JCV-non-permissive cells leads to an increase in JCV multiplication (Fig. 3c[Fig f3]).

The molecular regulation of JCV expression limits the range of cell types that can serve as sites of JCV latency, reactivation and virion multiplication (Messam *et al.*, 2003). The range of cells that support JCV expression is controlled, at least in part, by nucleotide sequences present in the viral promoter/enhancer (Amemiya *et al.*, 1992). While the JCV promoter contains binding sites for many nuclear transcription factors, the NF-1-binding site has shown to be critical for JCV multiplication (Amemiya *et al.*, 1992). Many JCV-susceptible cells showed higher levels of NF-1X protein expression, which implies the positive activation of JCV genes by NF-1X. Our observation of higher levels of expression of NF-1A protein in JCV-non-susceptible cells prompted us to manipulate NF-1A protein levels and to observe that a reduction in the level of NF-1A protein led to an increase in JCV multiplication.

Even though the four NF-1 genes are expressed in broadly overlapping patterns in different cell types, there are apparently unique mechanisms for expression of individual members of the NF-1 family. Since all NF-1 proteins bind to the same DNA binding region but differ in their promoter-specific activation and repression of transcription, the association of NF-1 proteins with the JCV DNA may be proportionate to the availability of an individual member of the NF-1 family. Our study shows that NF-1A and NF-1B proteins are abundant in JCV-non-susceptible progenitor cells. During the differentiation into JCV-susceptible PDA, the levels of NF-1A and NF-1B protein decreased significantly. Also, our anchored-JCV–promoter assay using the progenitor and PDA nuclear extract showed association of NF-1A, NF-1B or NF-1X in proportion to the level of protein (not shown). Hence, we assumed that binding of NF-1A or NF-1B from the (JCV-non-susceptible) progenitor cells at the JCV promoter might act as a transcriptional repressor of JCV-encoded gene expression. This is confirmed by our results, which also indicate that a reduction of the level of NF-1A protein in progenitor cells leads to susceptibility to JCV multiplication. Further, to extend an earlier report that TGF-*β*1 increased JCV multiplication in progenitor cells, we observed a decrease in NF-1A levels after TGF-*β*1 treatment. There was, however, no increase in the level of NF-1X protein.

Since the reduction of the level of NF-1A protein by siRNA led to an increase in NF-1B protein as well as JCV susceptibility, a proactive role of NF-1B was considered. However, a reduction of the level of NF-1B protein by siRNA was also associated with an increase in NF-1A and a subsequent decrease in JCV susceptibility. To overcome this reciprocal effect, we transfected progenitor cells with NF-1B-encoding plasmids, and found that overexpression of NF-1B protein did not lead to JCV multiplication (not shown). The reduction of levels of NF-1A protein during the differentiation of progenitor into PDA that leads to JCV susceptibility suggests that progenitor cells, with higher levels of NF-1A, experience a negative regulatory effect of NF-1A on JCV gene transcription. The promoters of several genes have been shown to be positively or negatively regulated by NF-1 protein; in particular, NF-1A-mediated transcriptional suppression of many genes has been reported (Wong *et al.*, 2007).

Furthermore, HeLa cells, which do not show JCV susceptibility, also express higher levels of NF-1A protein. Significantly, the addition of TGF-*β*1 resulted in JCV activity in HeLa cells, which do not normally support JCV activity. This increase in JCV activity was also associated with a decrease in NF-1A protein. Further, these results raise the possibility that, even though specific cellular receptors are important for JCV internalization, regulatory factors that act at the nuclear level are central to JCV multiplication. The observation that JCV-non-permissive cells, which express high levels of NF-1A, could also be infected with JCV by reducing the level of NF-1A protein supports the negative regulatory role of NF-1A on JCV multiplication. Our results show the important cell-specific regulatory mechanisms of NF-1A proteins in the control of JCV multiplication.

## Figures and Tables

**Fig. 1. f1:**
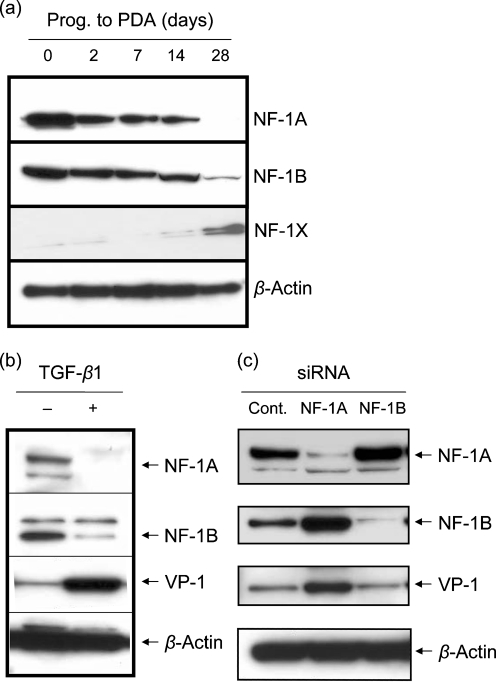
(a) Comparative Western blots utilizing anti-NF-1 protein antibodies with nuclear extracts of progenitors (Prog.) and PDAs at the time points indicated. Aliquots (5–7 μg protein equivalent) of nuclear extracts were resolved on 4–12 % gradient gels, transferred to PVDF membrane and probed with anti-NF-1A, anti-NF-1B, anti-NF-1X and anti-*β*-actin antibodies. *β*-Actin was a consistent immunoblot loading control for nuclear extracts. (b) Effect of TGF-*β*1 on the level of NF-1A protein in progenitors. Progenitors were exposed to JCV for 4 days in the presence or absence of TGF-*β*1. Aliquots (5 μg protein equivalent) of nuclear extracts were resolved on 4–12 % gradient gels, transferred to PVDF membrane and probed with anti-NF-1A, anti-NF-1B, anti-VP-1 and anti-*β*-actin antibodies. (c) Effect of NF-1 proteins on JCV multiplication. Progenitors were transfected with siNF-1A, siNF-1B or scrambled control siRNA (Cont.). At 16 h post-transfection, the culture medium was replaced with fresh medium containing JCV and, after 8 h exposure to JCV, the medium was removed by aspiration, cells were washed once and fresh medium was added. Four days after the transfection (3 days after JCV exposure), nuclear fractions were prepared for use in Western blot experiments with appropriate antibodies.

**Fig. 2. f2:**
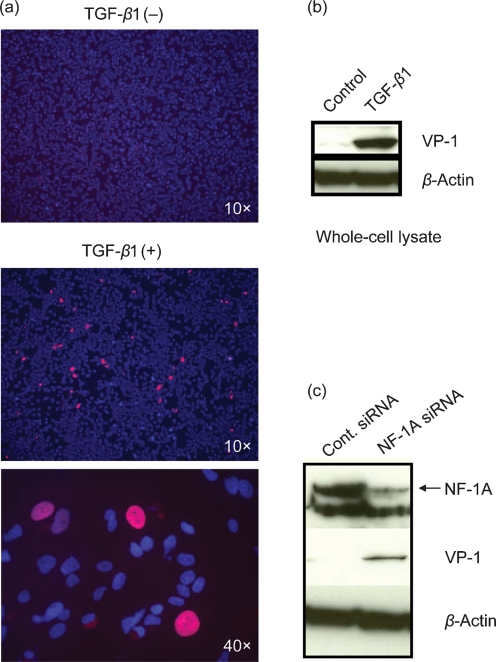
Effect of TGF-*β*1 on JCV multiplication in HeLa cells. (a) Immunostaining of HeLa cell cultures 4 days after JCV exposure and also with the addition of 5 ng TGF-*β*1 ml^−1^. Cells were fixed and permeabilized and then stained with anti-VP-1 (red) to determine relative JCV activity. Cellular nuclei were stained with DAPI (blue). (b) Western blots, from separate experiments under culture conditions identical to those used for immunostaining, of nuclear extracts that were resolved on 4–12 % gradient gels, transferred to PVDF membrane and probed with anti-VP-1 and anti-*β*-actin. (c) HeLa cells were transfected with NF-1A siRNA or with scrambled control siRNA (Cont.). At 16 h post-transfection, the culture medium was replaced with fresh medium containing JCV and, after 8 h of exposure to JCV, viral medium was removed by aspiration, cells were washed once and fresh medium was added. Four days post-transfection (3 days after JCV exposure), nuclear fractions were prepared for use in Western blot experiments with anti-NF-1A, anti-VP-1 and anti-*β*-actin antibodies.

**Fig. 3. f3:**
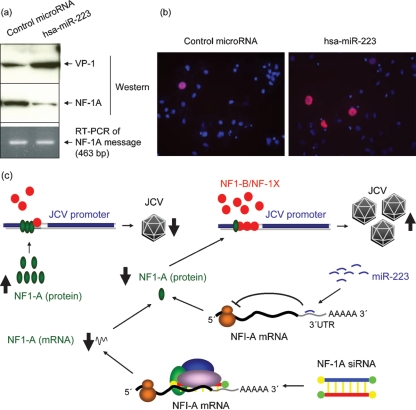
Effect of miR-223 on JCV multiplication. Progenitors were transfected with hsa-miR-223 or with control microRNA. At 16 h post-transfection, the culture medium was replaced with fresh medium containing JCV and, after 8 h exposure to JCV, viral medium was removed by aspiration, cells were washed once and fresh medium was added. (a) Four days post-transfection (3 days after JCV exposure), nuclear fractions were prepared for use in Western blot experiments with anti-VP-1, anti-NF-1A and anti-*β*-actin antibodies. (b) Four days post-transfection (3 days after JCV exposure), cells were fixed, permeabilized and stained with anti-VP-1 (red) and DAPI (blue). (c) Schematic representation of the relationship between the level of NF-1A and JCV multiplication. NF-1A siRNA decreases the level of NF-1A mRNA and thereby the level of protein. hsa-miR-223 acts on the 3′UTR of the NF-1A mRNA and decreases the level of NF-1A protein without affecting the level of mRNA. The presence of NF-1-class proteins leads to proportionate binding at the JCV promoter. Higher levels of NF-1X/B lead to an increase in NF-1X/B binding at the JCV promoter, thereby increasing JCV multiplication. The higher level of NF-1A leads to an increase in NF-1A at the JCV promoter, thereby decreasing JCV multiplication. Reducing the level of NF-1A protein by any means leads to an increase in JCV multiplication.
